# Stroke Analysis in Padel According to Match Outcome and Game Side on Court

**DOI:** 10.3390/ijerph17217838

**Published:** 2020-10-26

**Authors:** Jesús Ramón-Llin, José Guzmán, Rafael Martínez-Gallego, Diego Muñoz, Alejandro Sánchez-Pay, Bernardino J. Sánchez-Alcaraz

**Affiliations:** 1 Department of Musical, Plastic and Corporal Expression, University of Valencia, 46010 Valencia, Spain; jesus.ramon@uv.es; 2 Department of Physical Activity and Sport, Faculty of Sport Sciences, University of Valencia, 46010 Valencia, Spain; jose.f.guzman@uv.es (J.G.); rafael.martinez-gallego@uv.es (R.M.-G.); 3 Department of Musical, Plastic and Corporal Expression, Faculty of Sport Sciences, University of Extremadura, 10003 Cáceres, Spain; diegomun@unex.es; 4 Department of Physical Activity and Sport, Faculty of Sport Sciences, University of Murcia, 30720 San Javier, Spain; bjavier.sanchez@um.es

**Keywords:** performance analysis, racquet sports, professional sport, game actions

## Abstract

The aim of this study was to analyze the distribution of padel strokes, their effectiveness, direction, and court zone, comparing between the winning and losing pairs in the match and the playing side of the players. The sample included 8441 strokes corresponding to 1055 points out of a total of nine padel matches in the First National Category. The variables analyzed were type of stroke, court area, effectiveness and directions of the strokes, match outcome, and game side. Matches were analyzed through systematic observation. The results showed that the winning pair made a significantly higher percentage of winners, and cross-court smashes and volleys from the offensive zone. In addition, players on the left side executed a higher percentage of cross-court and winning shots than the players on the right side. Such knowledge may constitute a useful guide in the design of appropriate game strategies and specific training sessions based on the shots that will help players to win the match according to the role of the player and depending on their game side.

## 1. Introduction

Padel is a relatively new sport [1], which is practiced in pairs (2 vs. 2) on a 20 × 10 m court, surrounded by walls or glass and metal fences, which allow the bounce of the ball, and is scored like tennis [2]. It is characterized as a sport with an average duration of efforts between 10–15 s per point [3,4], different technical-tactical actions, and high-intensity movements from the players in different directions, which require continuous decision making [5]. In recent years, there have been numerous studies on padel that have evaluated parameters related to performance analysis [6], with the aim of extracting relevant data from spontaneous behaviors and in real contexts of competition [7]. These investigations provide objective information on real game situations [8], which is vital for planning specific and effective training [9], designing strategies for better performance, and improving decision making and feedback, according to the behavior of the players during the game [10].

These investigations have been based on those variables or indicators that contribute to success in competition and that are common in racquet sports [11]. In this respect, studies carried out with professional padel players have determined that, from a tactical point of view, there are two basic game positions: The attack position, which is when the pair plays close to the net, and the defense position, which is where the pair plays at the baseline of the court [12,13]. This approach has been confirmed by different studies that concluded that there is a greater probability of winning the point when occupying positions close to the net [14,15]. These investigations have shown that more than 80% of the padel winning points are made from the attack position, using different strokes such as volleys (20–25%), the tray, and the smash (12–18%) [16,17,18]. On the other hand, players in defensive positions perform other types of strokes, among which the lob predominates, with the aim of forcing the attacking pair to move backwards to hit the ball in positions further away from the net or sending them to the baseline in order to reach the offensive position [19].

Therefore, the players constantly try to get a position close to the net, for which they use different behaviors and technical-tactical actions, which define different styles of play [20,21]. The distribution of the different types of stroke, their trajectories, and their efficacy stand out among these behaviors [16,22,23]. The results of the studies have shown that these variables may differ depending on the gender, laterality, or level of the players [17,24]. Particularly in padel, the optimal use of the space is essential to enhance performance and increase scoring rate [20]. Results reveal a solid structure of padel game dynamics depending on game side on court. Left-side players seems to be more effective using smashes but made more errors when hitting the ball after bouncing on the wall; in turn, right-side players made more lobs and committed fewer errors [20]. This seems to indicate a specialization of left-sided as ‘scorers’ and right-sided as ‘defenders’ [25]. In this sense, a better knowledge on players’ profiles regarding playing side (right or left) is required to set optimal training plans and goals. However, there is a lack of studies that identify the different actions that players perform comparing winners and losers in the match or the side of the court on which the padel player plays. Knowledge of these parameters will help to achieve greater specialization in training sessions, prioritizing those actions that will enable success in the match, and differentiating according to the side of the player depending on the court. In this sense, we hypothesized that paddle players will have different tactical and technical actions according to position and performance. Therefore, the main aim of this study was to analyze the distribution of the different strokes, their effectiveness, direction, and hitting area, and to compare these data according to the final result of the match and the playing side of each player.

## 2. Materials and Methods

### 2.1. Sample and Variables

The sample included 8441 shots corresponding to 1055 points from nine matches (three finals and six semifinals) from a total of three top national tournaments (First National Category). A total of 24 male padel players (mean (SD) age: 31.18 (7.27) years; height: 181.3 (4.1) cm) performed the matches.

**Figure 1:**
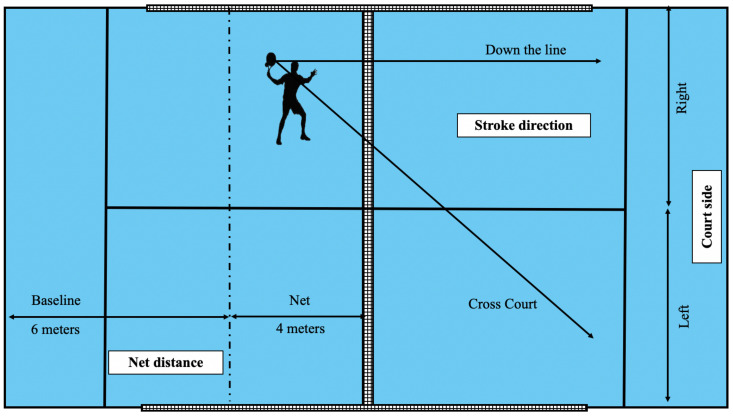
Net distance (baseline and net), playing side (right and left), and stroke trajectory (down the line or cross court).

The matches were played following the official game regulations [2]. The ethics board of the local university reviewed and approved the study (ethic code: 154/2020). The following variables were analyzed:

- Type of stroke: The technical actions of hitting were analysed distinguishing between [14] serve (first or second serve), volleys (stroke without a bounce that is made by hitting the ball at head height, with either a forehand or backhand), tray (stroke without a bounce that is made by the dominant side of the player, hitting the ball at an intermediate height between the volley and the smash and with a slice effect), smash (shot without a bounce that is made by the dominant side of the player, hitting the ball with the arm outstretched, over the head, with a flat or topspin effect), ground stroke (forehand or backhand direct shot), back-wall stroke (forehand or backhand after a rebound on the back wall), side-wall stroke (forehand or backhand after a rebound on the side wall), double-wall stroke (forehand or backhand after a bounce on two walls of the court, depending on the bounce order (side and back wall or back and side wall)), lob (stroke made with a high trajectory with the aim of overcoming the opponents that are at the net), and wall boast (forehand or backhand hitting the ball against the wall of the court itself).
- Court area: Two areas were distinguished, the net area (offensive area) and the baseline area (defensive area). The line that delimited both areas was located on the visual reference on the horizontal-side fence of the court, four meters from the net (Figure 1), following a previous proposal [10,26].
- Stroke direction: Direction was divided between two possible options, down the line and cross court (Figure 1).
- Stroke effectiveness: The stroke effectiveness classification distinguished between continuity (shot causing the point to continue), winner (the player wins the point with a direct stroke), and error (the player loses the point by missing the shot) [7].
- Playing side: The player on the left and right sides of the game was distinguished in each pair (Figure 1).

### 2.2. Procedure

Firstly, informed consent was requested from tournament organizers and athletes for the recording of matches. Two digital Bosch Dinion Model IP 455 video cameras (Bosch, Munich, Germany) were used to film the matches (25 frames per second), sagittally placed over the courts at 6 m from the center and over the service line. The techniques for transferring video images into Tracker were identical to SAGIT/Squash, i.e., automatic processing with operator supervision, which has been well documented [27]. This software allows to track the movements of the players automatically. Similarly, the reliability for the resultant calculations of distance and speed for each player and positions on court has been shown to be acceptable for analysis purposes [28]. The data were recorded through systematic observation, using specific software for video analysis: LINCE software [29]. The Kinovea software (V.08.26, www.kinovea.org, Bordeaux, France) was used to place a visual grid over the video image for court side, net distance, and stroke direction. Two observers, graduates in Physical Activity and Sports Sciences, and padel coaches, with more than 10 years of experience in the sport, were specifically trained for this task. The training focused on the clear identification of the variables and the use of the observational instrument software (Lince and Kinovea). At the end of the training process, each observer analyzed the same two sets in order to calculate the inter-observer reliability with the Multirater Kappa Free [30], obtaining values above 0.80. To ensure the consistency of the data, intra-observer reliability was evaluated at the end of the observation process, obtaining minimum values of 0.80. The kappa values showed the degree of agreement as very high (> 0.80) [31].

### 2.3. Data Analysis

The data were obtained via the visual analysis of matches. These data were entered onto a spreadsheet (Microsoft Excel) for processing purposes. From the spreadsheet, the data were exported to the IBM SPSS 25.0 statistical package for Macintosh (IBM Corp: Armonk, NY, USA) for analysis. Firstly, a descriptive exploration of the data obtained was carried out and frequency (*n*) and percentage (%) were calculated. Subsequently, the Kolmogorov–Smirnov tests were performed for the study of normality and the Levene test for the homogeneity of variances. A comparison was made of the statistics on the type of strokes, efficiency, and direction according to match outcome and the playing side using Pearson’s chi-square test. In the variables of type of stroke and effectiveness of the stroke subsequent Z tests were carried out to compare column proportions, adjusting the values of *p* < 0.05 according to Bonferroni. The associations among the categories of the variables was performed with corrected standardized residuals (CSR). The effect size was calculated from Crammer’s V [32]. The Crammer’s V effect size was interpreted as small, medium, and large according to degrees of freedom [33]. A significance level of *p* < 0.05 was established.

## 3. Results

Table 1 shows the descriptive results of the distribution of the different strokes, their effectiveness, and direction, comparing the winning and losing pair of the match. The type of hitting performed by both pairs showed significant differences according to match outcome (χ^2^ = 77.19; *gl* = 11; *p* < 0.001; *V* = 0.096). The winners made a significantly higher percentage of smashes and trays and a lower number of side-wall shots, side and back wall, and wall boast than the losers. The effectiveness of the stroke also showed significant differences among pairs (χ^2^ = 16.579; *gl* = 2; *p* < 0.001; *V* = 0.044), with the winning pair making a higher percentage of winners than the losers. Moreover, regarding the direction of the shots, significant differences were found between winners and losers (χ^2^ = 7.306; *gl* = 1; *p* = 0.007; *V* = 0.033), with the winning pair in the match performing a higher percentage of cross-court shots than down the line.

**Figure 2:**
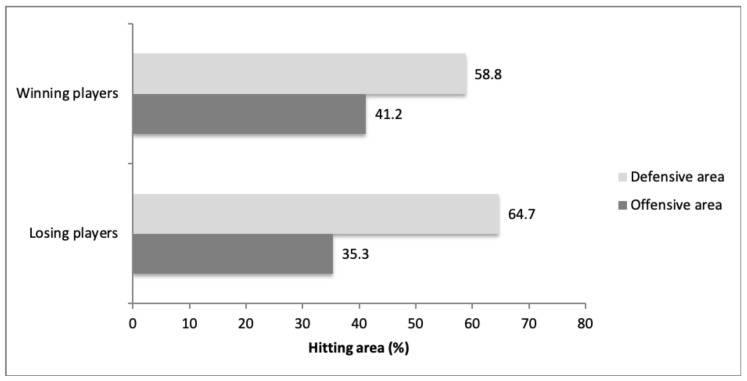
Distribution of the percentage of defensive and offensive shots performed by the winning and losing pairs.

The results relative to the distribution of the strokes according to the area of the court are shown in Figure 2. The area of the court where the shots were made was significantly different according to match outcome (χ^2^ = 31.145; *gl* = 1; *p* < 0.001; *V* = 0.060). Thus, the winning players made a higher percentage of shots in offensive areas (41.2%) than the losing players (35.3%).

Table 2 shows the descriptive results of the distribution of the different strokes, their effectiveness, and direction, depending on the playing side. The player on the left side made a significantly higher number of hits than the player on the right side (χ^2^ = 42.588; *gl* = 1; *p* < 0.001). The playing side of the players also showed significant differences in the type of stroke (χ^2^ = 57.895; *gl* = 11; *p* < 0.001; *V* = 0.084). Thus, the players on the left side performed a higher percentage of trays, smashes, side-wall, and wall boast shots than the players on the right side. In addition, differences were found in the effectiveness of these strokes depending on the side of the court (χ^2^ = 17.375; *gl* = 2; *p* < 0.001; *V* = 0.045). The players on the left side made a significantly higher percentage of winners and a lower percentage of errors than the players on the right side. Finally, significant differences were also found in the shot directions (χ^2^ = 13.878; *gl* = 1; *p* < 0.001; *V* = 0.043) with the players on the left side making a higher percentage of cross-court and fewer down-the-line shots than the players on the right side.

**Figure 3:**
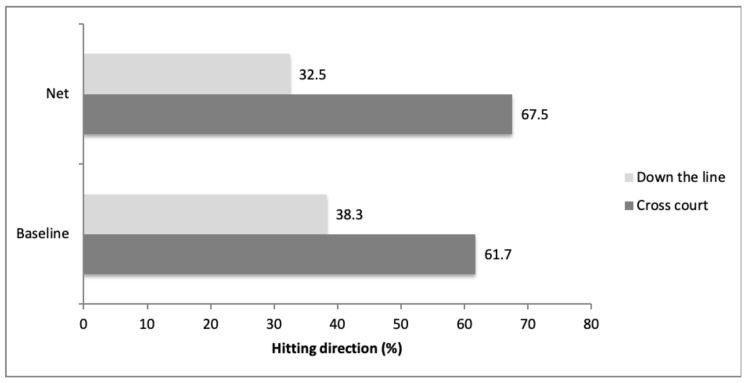
Distribution of the percentage of down-the-line and cross-court shots according to the court area.

The results related to the direction of the strokes in each court area are shown in Figure 3. The area of the court where the strokes were made significantly influenced the direction of the strokes made by the padel players (χ^2^ = 29.415; *gl* = 1; *p* < 0.001; *V* = 0.058). Thus, when the players hit in offensive positions, close to the net, they made a higher percentage of cross-court shots (67.5%) than when they were positioned at the baseline of the court (61.7%).

## 4. Discussion

The aim of the present study was to analyze the distribution of the different strokes, their effectiveness, direction, and hitting area, and compare them according to the final result of the match and the playing side of each padel player. The results showed that the most-used strokes by the players were volleys, ground strokes, and back-wall strokes, results similar to those of other studies that have quantified the distribution of padel strokes [16,17,18,23]. However, these results may be especially relevant when analyzed according to the result of the match, since they would show the strokes that are most used to win a padel match. In this regard, the results of this study indicated that the winning pairs perform a significantly higher percentage of smashes and volleys and a lower number of ground strokes, walls strokes, and lobs than the losers.

Considering the area of the court where the stroke is made, the results showed that the winning players made a significantly higher percentage of shots in positions close to the net, results that confirm the data already provided by similar studies [10,14]. Additionally, the winning pairs performed a higher percentage of cross-court shots than the losing pairs. These results confirm the importance of cross-court shots in professional padel [34,35]. The use of cross-court shots would send the ball toward the side of the court that could cause the ball to rebound off the metal fence, the side wall, or the corner between the back wall and the side wall, increasing the opponent’s uncertainty and the chance of their making mistakes.

Thus, the results of this study also showed that the players who lost the match made a higher percentage of shots after the rebound on the side wall or double wall, confirming the data from other studies that observed that the players on the baseline perform a greater number of strokes at the corners of the court [14]. Thus, it seems that varying the directions of the shots and hitting the ball to the corners of the court have been two of the fundamental tactical principles to achieve success in racket sports [5,19,36]. Considering the effectiveness of the stroke, the winning pairs achieved a higher percentage of winners (5.6%) and a lower percentage of errors (7.5%) than the losers, which has been corroborated in other studies [10], and indicated that most of the winners were made through smashes and trays in areas close to the net [14,34]. However, deeper analysis and complementary variable collection is encouraged for a more relevant advance in this knowledge.

One of the main contributions of this study is the distribution of strokes regarding the side of the court on which the player is playing. The results showed that the left-side player performs more strokes than the right-side player, and there are even differences in the distribution of strokes. In this regard, the left-side players made a significantly higher percentage of trays and smashes than the players on the right side (Table 2). The smash seems to be the stroke with the highest percentage of efficiency in padel at the professional level [37]. The data from this study confirm a greater specialization of the players on the left side in the winners, which would define them as having a more offensive style of play. These results could be related, also, to the laterality of the players, since, in pairs with two right-handed players, the player on the left side is the one who would perform the most power smashes, being able to hit the ball with his dominant arm in the central area of the court [20]. So, if the pair of players included two right-handed players, the one on the right side has the paddle near the side wall, which sometimes constitutes a limiting factor when returning a ball. However, a game combination including a left-handed players on the right side allows both players defending the center line in better conditions (i.e., both using quick and balanced forehand strokes) and making easy the use of overhead strokes for returning balls near the side wall [19,20,25]. Their greater participation would be directly related to the ability to finish points. In addition, the results obtained showed a higher percentage of the use of cross-court shots in the players on the left side, trajectories that have shown greater effectiveness in this study.

The results of this study present some limitations that must be considered when interpreting them. In the first place, the laterality of the players has not been taken into account, a variable that could influence the distribution, trajectory, or effectiveness of the strokes [20]. Furthermore, the research sample has only evaluated high-level male players, so it is proposed that future studies compare these data in other categories such as women or young players. Finally, stroke effectiveness distinguished winners, errors, and continuity, but continuity could mean different realities, from easy balls to very difficult balls, so it would be recommended to separate in future studies.

## 5. Conclusions

This study presents new contributions on game analysis indicators in national padel level. The data show that the winning pairs in padel execute a higher percentage of volleys, trays, and smashes, predominantly cross court and with fewer errors. Moreover, the player on the left side of the court makes more total shots per match and a significantly higher percentage of smashes and cross-court shots than the player on the right side. These data have an important practical application, since they will allow padel coaches and sports technicians to design exercises by selecting those strokes and directions that will lead to success in the match, adapting the tasks specifically to the two players in the pair, differentiating between the right- and left-side players’ style of play. The findings of this study suggest that coaches should consider teaching cross-court volleys and smashes from a tactical perspective, because controlling the net game seems to be a key factor in national padel that may distinguish the best players.

## Figures and Tables

**Table 1:** Type of stroke, effectiveness, and direction according to match outcome.

	Winning			Losing			Sig.
	*N*	%	*CSR*	*N*	%	*CSR*	
**Type of stroke**							
Serve	490	11.6	−1.1	525	12.5	1.1	
Volley	1133	26.8	2.0	1056	25.0	−2.0	
Tray	368	8.7a	4.1	255	6.0b	−4.1	
Smash	246	5.8a	3.8	185	4.4b	−3.8	
Ground stroke	655	15.5	−0.8	653	15.5	0.8	
Back wall	528	12.5	−0.6	540	12.8	0.6	<0.001
Side wall	209	4.9a	−3.4	272	6.5b	3.4	
Side and back wall	86	2.0a	−2.1	112	2.7b	2.1	
Back and side wall	79	1.9	−2.9	106	2.5	2.9	
Lob	378	8.9	−1.8	391	9.3	1.8	
Wall boast	53	1.2a	−3.5	121	2.9b	3.5	
**Effectiveness of stroke**							
Continuity	3671	86.9	−2.3	3716	88.1	2.3	
Winner	236	5.6a	4.3	158	3.7b	−4.3	<0.001
Error	318	7.5	−1.2	342	8.1	1.2	
**Direction**							
Down the line	1464	34.7	−2.0	1580	37.5	2.0	0.007
Cross court	2761	65.3	2.0	2636	62.5	−2.0	

Note: *N* = frequency; % = percentage; CSR = Corrected Standardized Residuals; a,b = indicate significant differences in the *Z* tests for comparison of column proportions from *p* < 0.05 adjusted according to Bonferroni.

**Table 2:** Differences in the number and type of strokes, their effectiveness, and direction depending on the playing side of the court.

	Right Side Player			Left Side Player			Sig.
	*N*	%	*CSR*	*N*	%	*CSR*	
**Participation**							
Number of shots	3934	46.60	−4.1	4507	53.40	4.1	<0.001
**Type of stroke**							
Serve	480	12.2	1.1	536	11.9	−1.1	<0.001
Volley	1078	27.4	3.5	1111	24.7	−3.5	
Tray	266	6.8a	−1.6	357	7.9b	1.6	
Smash	148	3.8a	−4.2	282	6.3b	4.2	
Ground stroke	634	16.1	1.6	674	15.0	−1.6	
Back wall	524	13.3	0.3	544	12.1	−0.3	
Side wall	198	5.0a	−1.2	283	6.3b	1.2	
Side and back wall	86	2.2	−0.2	112	2.5	0.2	
Back and side wall	75	1.9	−1.0	110	2.4	1.0	
Lob	380	9.7	1.1	389	8.6	−1.1	
Wall boast	65	1.7a	−0.2	109	2.4b	0.2	
**Effectiveness of stroke**							
Continuity	3484	88.6	0.8	3904	86.6	−0.8	<0.001
Winner	143	3.6	−3.2	250	5.5	3.2	
Error	307	7.8	1.9	353	7.8	−1.9	
**Direction**							
Down the line	1501	38.1	2.3	1543	34.2	−2.3	<0.001
Cross court	2433	61.9	−2.3	2964	65.8	2.3	

Note: *N* = frequency; % = percentage; CSR = Corrected Standardized Residuals; a,b = indicate significant differences in the *Z* tests for comparison of column proportions from *p* < 0.05 adjusted according to Bonferroni.
